# Quantification of the sit-to-stand movement for monitoring age-related motor deterioration using the Nintendo Wii Balance Board

**DOI:** 10.1371/journal.pone.0188165

**Published:** 2017-11-14

**Authors:** Go Yamako, Etsuo Chosa, Koji Totoribe, Yuu Fukao, Gang Deng

**Affiliations:** 1 Organization for Promotion of Tenure Track, University of Miyazaki, Miyazaki, Japan; 2 Department of Medicine of Sensory and Motor Organs, Division of Orthopedic Surgery, Faculty of Medicine, University of Miyazaki, Miyazaki, Japan; 3 Department of Mechanical Design Systems, Faculty of Engineering, University of Miyazaki, Miyazaki, Japan; University of Memphis, UNITED STATES

## Abstract

Simple methods for quantitative evaluations of individual motor performance are crucial for the early detection of motor deterioration. Sit-to-stand movement from a chair is a mechanically demanding component of activities of daily living. Here, we developed a novel method using the ground reaction force and center of pressure measured from the Nintendo Wii Balance Board to quantify sit-to-stand movement (sit-to-stand score) and investigated the age-related change in the sit-to-stand score as a method to evaluate reduction in motor performance. The study enrolled 503 participants (mean age ± standard deviation, 51.0 ± 19.7 years; range, 20–88 years; male/female ratio, 226/277) without any known musculoskeletal conditions that limit sit-to-stand movement, which were divided into seven 10-year age groups. The participants were instructed to stand up as quickly as possible, and the sit-to-stand score was calculated as the combination of the speed and balance indices, which have a tradeoff relationship. We also performed the timed up and go test, a well-known clinical test used to evaluate an individual’s mobility. There were significant differences in the sit-to-stand score and timed up and go time among age groups. The mean sit-to-stand score for 60s, 70s, and 80s were 77%, 68%, and 53% of that for the 20s, respectively. The timed up and go test confirmed the age-related decrease in mobility of the participants. In addition, the sit-to-stand score measured using the Wii Balance Board was compared with that from a laboratory-graded force plate using the Bland–Altman plot (bias = −3.1 [ms]^-1^, 95% limit of agreement: −11.0 to 3.9 [ms]^-1^). The sit-to-stand score has good inter-device reliability (intraclass correlation coefficient = 0.87). Furthermore, the test–retest reliability is substantial (intraclass correlation coefficient = 0.64). Thus, the proposed STS score will be useful to detect the early deterioration of motor performance.

## Introduction

Aging is an inevitable human condition that is mainly characterized by irreversible biological changes, such as reduction in muscle mass and strength, loss of mobility and balance, and impairment of motor coordination [1, 2]. This physiological decline is typically observed in elderly people and frequently leads to difficulties in performing activities of daily living (ADLs), and consequently, a decrease in quality of life. However, as the average life expectancy increases, the desire of people to live independently has also increased, leading to significant interest in the development of quantification methods for easy measurement of functional motor performance over a wide age range, with the aim of preventing difficulties in performing ADLs.

The sit-to-stand (STS) movement, in which a person transitions from a sitting to a standing position, is an important and frequently performed task. Among all motor tasks associated with ADLs, the STS movement has been identified as the most difficult and mechanically demanding because it requires significant leg muscle strength, a wide range of joint movement, and good balance control [3, 4]. In particular, the magnitude of moments at the hip joint is greater during the STS movement than during climbing stairs or walking [4] and balance is required to maintain equilibrium as the body mass is transferred from the chair seat to the feet. Accordingly, age-related decreases in muscle strength and balance control in elderly people are frequently associated with difficulties in completing STS movements [5, 6]. An inability to perform this can limit a person’s independence and lead to impaired functioning and mobility in ADLs, resulting in institutionalization or even death [7]. A decreased ability to perform a STS movement, particularly in a rapid manner, has also been associated with increased risks of falling [8] and hip fracture in elderly people [9]. To date, many researchers have investigated the mechanics of the STS movement in healthy young and elderly people [10, 11], as well as in people with various pathologies, such as stroke [12], hemiplegia [13], Parkinson’s disease [14], obesity [15], total hip arthroplasty [16], and muscle damage [17]. The STS movement has been identified as a valuable source of information regarding an individual’s motor performance abilities [18–22].

We hypothesized that motor performance could be estimated using the ground reaction force and center of pressure (COP) trajectory data obtained from the Nintendo Wii Balance Board (WBB; Nintendo, Kyoto, Japan) during the STS movement. WBB is designed to serve as a video game controller that is increasingly used for assessment of postural control in rehabilitation [23–28]. Using data from WBB, we defined a new index, the STS score, as a representative of STS movement to estimate individual motor status. The present study investigated age-related changes in the STS score. To further confirm the age-related decline of mobility, we performed the timed up and go (TUG) test, a well-known clinical test.

## Materials and methods

### Participants

A total of 503 healthy participants (age, 20–88 years; male/female ratio, 226/277) were recruited from our university, private and public corporations, and local senior citizen clubs (*rojin* clubs in Japanese). Professional athletes were not included as participants. None of the participants had any known musculoskeletal or neuromuscular conditions that would limit their mobility or their ability to perform the STS movement. The ethical committee of University of Miyazaki approved the protocol for the study (reference number: 2014–231) and all participants provided written informed consent. The participants were divided into seven 10-year age groups. The demographic data in each age group is shown in Table 1.

**Table 1:** Demographic data for each 10-year age group of participants.

Age group (years)	n (Male/Female)	Age (years)	Height (cm)	Weight (kg)	BMI (kg/m^2^)
**20–29**	94 (36/58)	25.1 ± 2.7	163.5 ± 9.1	56.9 ± 12.1	21.1 ± 3.0
**30–39**	82 (37/45)	34.7 ± 3.1	164.5 ± 8.5	59.6 ± 11.5	21.9 ± 3.1
**40–49**	81 (40/41)	44.2 ± 2.9	164.5 ± 7.6	61.9 ± 11.6	22.8 ± 3.6
**50–59**	70 (41/29)	54.2 ± 2.9	164.5 ± 8.3	63.2 ± 12.1	23.3 ± 3.5
**60–69**	46 (21/25)	64.1 ± 2.7	158.7 ± 9.4	57.1 ± 9.4	22.6 ± 3.1
**70–79**	85 (34/51)	75.0 ± 2.9	153.3 ± 7.7	56.1 ± 9.3	23.8 ± 3.1
**80–89**	45 (17/28)	82.6 ± 2.2	152.2 ± 9.7	53.7 ± 9.0	23.2 ± 3.1
**Total**	503 (226/277)	50.9 ± 19.7	160.8 ± 9.8	58.6 ± 11.3	22.6 ± 3.4

Values are presented as mean ± standard deviation.

### Instructions regarding the STS movement

**Fig 1:**
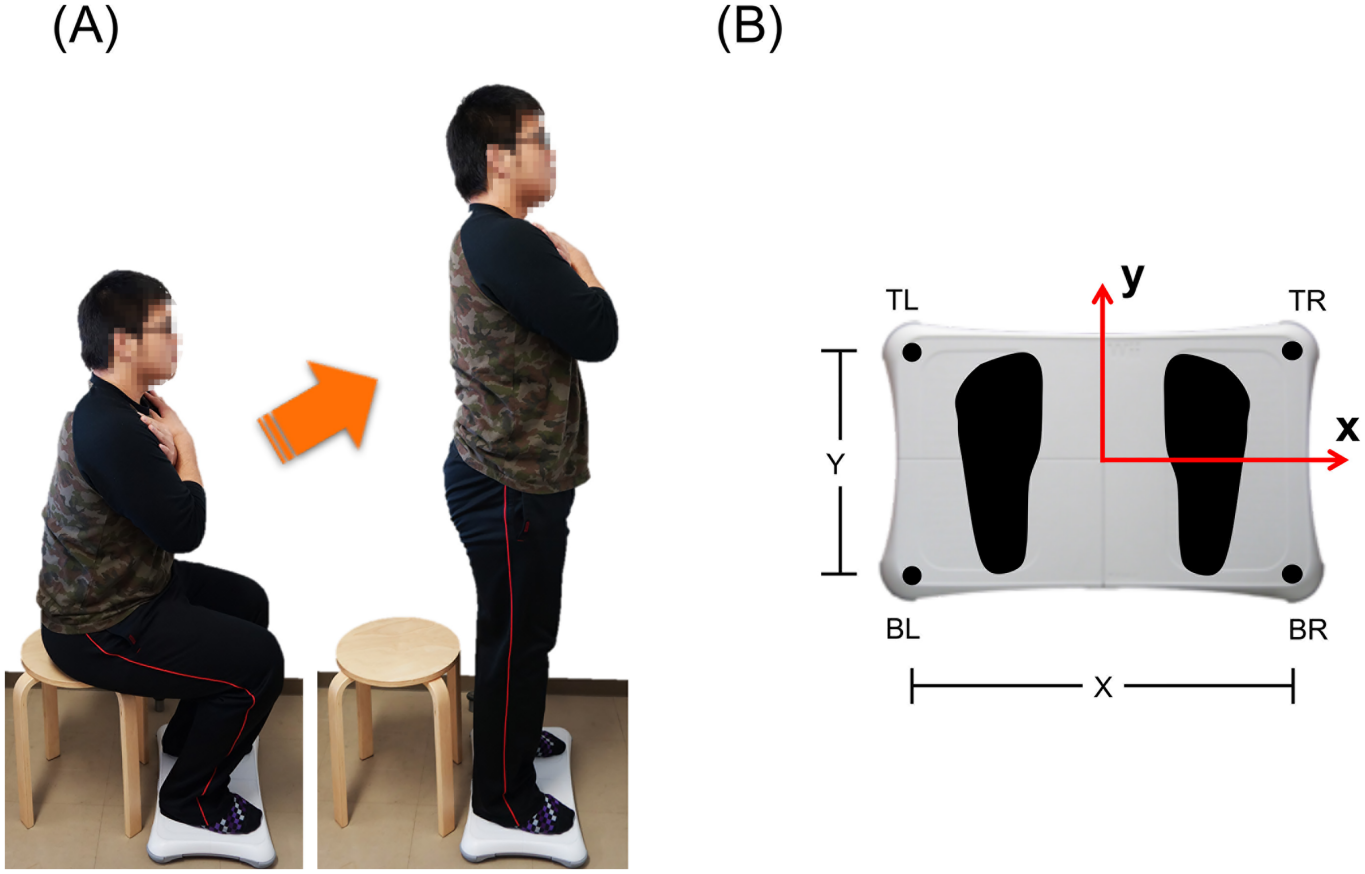
Sit-to-stand testing. (A) WBB is placed under feet. (B) Coordinate system. Force transducers are placed in each corner of WBB: top right (TR), top left (TL), bottom right (BR), and bottom left (BL). The distances of each transducer are 43.3 cm for x-direction (X) and 22.8 cm for y-direction (Y).

The participants were instructed to stand up from a chair as rapidly as possible in a safe manner, immediately recover balance, and stand as still as possible in an upright posture for 5 s. To perform this test, WBB was placed beneath the feet, and participants were seated on an armless, backless chair. The seat height was adjusted to the participant’s knee joint line by using 2-cm-thick wooden boards. The feet were placed at shoulder-width apart on WBB in 20° dorsiflexion (Fig 1A). The participants crossed their arms on their chest during testing. Before data were recorded, each subject was allowed one opportunity to practice the procedure. Each subject performed two trials separated by a 1-min interval. We did not observe any accident, such as falling, during this procedure.

### Data acquisition and processing for quantification of the STS movement

WBB, which consists of a rigid platform with four strain gauge-based vertical load transducers located in the feet at each corner (Fig 1B), was used to calculate the ground reaction force and COP. During the STS movement, four force data were streamed to a laptop computer using a Bluetooth HID wireless protocol and custom programs written in C# and were sampled at approximately 100 Hz. [29]. The sampling rate was inconsistent; therefore, the data were resampled at 100 Hz using a linear interpolation.

The four forces (*F*_*TR*_, *F*_*TL*_, *F*_*BR*_, and *F*_*BL*_) obtained from each transducer of WBB were processed to calculate the vertical ground reaction force and COP using a custom MATLAB program (R2014b, MathWorks, Natick, RI, USA). The vertical ground reaction force and COP (C_x_, C_y_) were represented as the following equations using the center of WBB as the origin and the dimensions of X and Y:

$$Vertical\;ground\;reaction\;force\;[kgf]\;=\;F_{TR}+F_{TL}+F_{BR}+F_{BL}$$

$$C_{x}[cm]\;=\;\frac{X}{2}\frac{\left(F_{TR}+F_{BR})-(F_{TL}+F_{BL}\right)}{F_{TR}+F_{BR}+F_{TL}+F_{BL}}$$

$$C_{y}[cm]\;=\;\frac{Y}{2}\frac{\left(F_{TR}+F_{TL})-(F_{BR}+F_{BL}\right)}{F_{TR}+F_{BR}+F_{TL}+F_{BL}}$$

**Fig 2:**
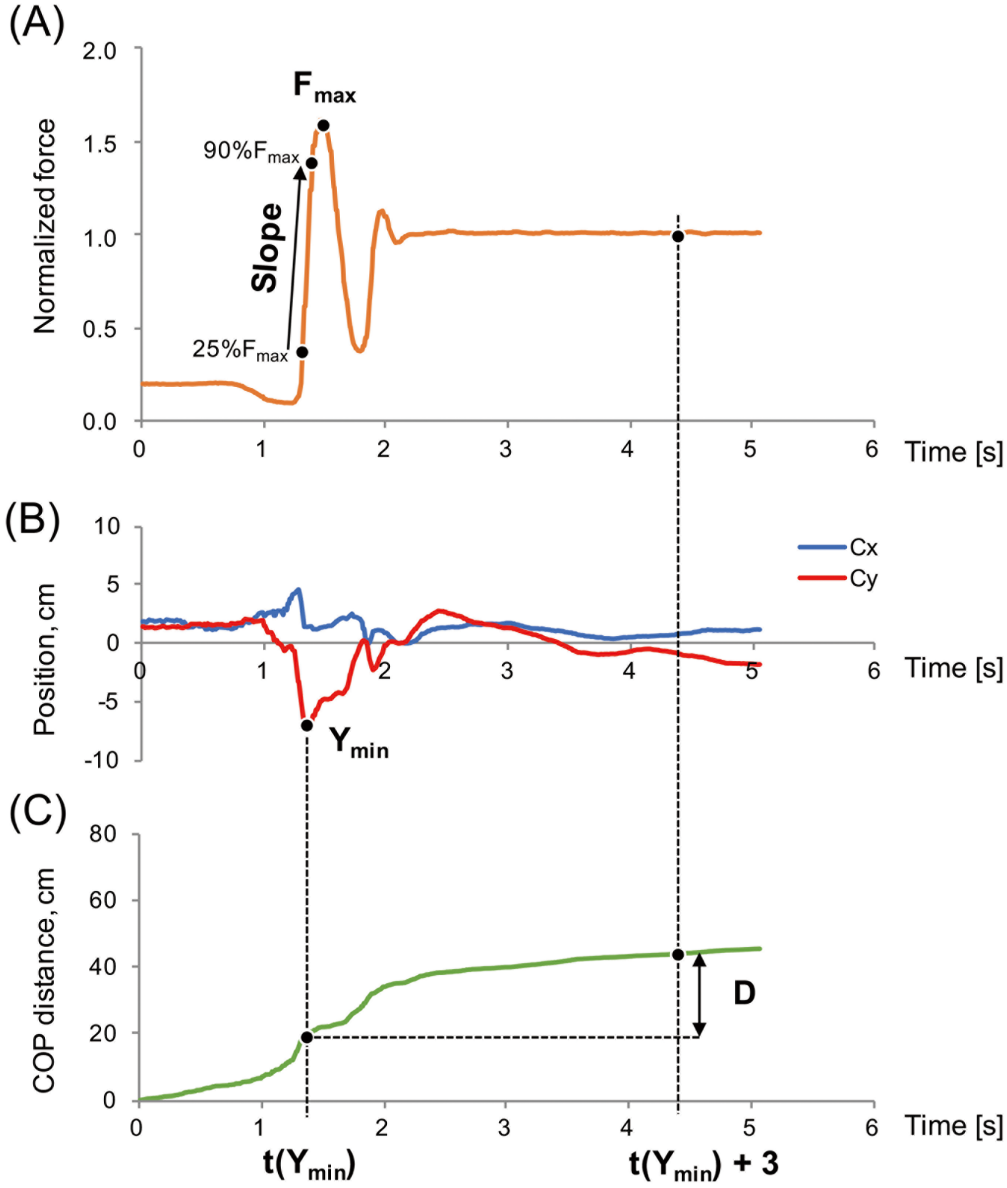
Graphs illustrating the force plate-based data. (A) Vertical ground reaction force; (B) position of center of pressure (COP); Cx, right direction, Cy, anterior direction; (C) total COP distance.

Subsequently, the vertical ground reaction force was normalized to the participant’s body weight (Fig 2A), and the COP trajectory distance was calculated using COP positional data (C_x_, C_y_; Fig 2B and 2C).

In this study, we defined a novel index using the calculated STS movement-related speed and balance indices. The index for speed (speed score, s^-1^), which represents the speed of the STS movement, was defined as the slope of the normalized force–time curve and calculated using the linear portion of the curve as 65% of the maximum normalized force (*F*_*max*_) divided by the time period between the time point at 25% of *F*_*max*_ (*t*_*25%Fmax*_) and that at 90% of *F*_*max*_ (*t*_*90%Fmax*_) (1).

$$Speed\;score\;[s^{-1}]\;=\;slope\;=\;\frac{0.65\;\times \;F_{max}}{t_{90\%F_{max}}\;-{\;t}_{25\%F_{max}}}$$ (1)

The index for balance (balance score, m^-1^), which represents balance control during the STS movement and is based on the COP trajectory distance (*D*, m), was defined as the inverse of *D* from the time when *Cy* was minimized [*t(y*_*min*_*)*] to +3 s (2). The time *y*_*min*_ indicates the time of lift-off from the chair. At 3 s from the time *y*_*min*_, the COP trajectory distance was substantially stable, which indicates that the upright posture of the participants was stabilized.

$$Balance\;score\;[m^{-1}]\;=\;D^{-1}\;=\;{\left\{D[t(y_{min})\;+\;3]\;-\;D[t(y_{min})]\right\}}^{-1}$$ (2)

The above two indices were then used to define the novel STS score (ms)^-1^ as the product of the speed and balance scores, with the intention of quantifying STS movement performance (3). Higher STS scores indicate better performance. These two indices have a tradeoff relationship, because, generally, if the movement speed (speed score) increases, it becomes difficult to control balance and remain as still as possible (balance score).

$$STS\;score\;[{\left(ms\right)}^{-1}]\;=\;Speed\;score\;\times \;Balance\;score$$ (3)

These indices were calculated from each set of trial data using the MATLAB program. For each participant, a practice trial was followed by two timed trials, and the highest trial of the STS score was selected as a result for further analysis.

### Agreement and test–retest reliability

For preliminary study, we evaluated the agreement of the above-defined indices calculated from WBB. The indices were compared with those obtained using a laboratory-graded force plate. Another five participants (mean age, 22.6 ± 1.5 years; height, 1.67 ± 0.12 m; weight, 66.0 ± 23.1 kg) were recruited and asked to perform three STS movement trials each. WBB was placed on a force plate (FP; AMTI, model OR6; 60 × 40 cm). During STS movement, force data were collected by both WBB and FP devices simultaneously. Data from FP were sampled at 1000 Hz and filtered using a fourth order Butterworth filter with a low pass cut-off frequency of 300 Hz. Then, the data were resampled at 100 Hz, and the indices were calculated using the MATLAB program. For each index, we examined the agreement between WBB and FP using the Bland–Altman plot [30] and intraclass correlation coefficient (Two-way random single measures, ICC_(2,1)_).

In addition, to determine test–retest reliabilities of the indices, intraclass correlation coefficients (One-way random single measures, ICC_(1,1)_) were calculated among another 24 young participants (mean age, 22.3 ± 1.4 years; height, 1.71 ± 0.08 m; weight, 66.9 ± 12.3 kg), who were tested repeatedly at 3-h intervals.

### TUG test procedure

The TUG test was developed to improve evaluations of functional performance and mobility [31]. This test measured the time it took a participant to rise from a chair, walk 3 m, turn around, walk back, turn around, and sit down again. Shorter TUG time indicates better performance. Participants were instructed to walk as quickly and safely as possible. For each participant, a practice trial was followed by two timed trials, and the fastest trial was selected for further analysis.

### Statistical analysis

Statistical analyses were performed using SPSS 22.0 (IBM SPSS, Chicago, IL, USA). Each index was compared among the 10-year age groups using one-way ANOVA followed by Dunnett’s T3 multiple comparison test. A P value of <0.05 was considered statistically significant.

## Results

### Typical graph patterns of young and elderly participants in vertical reaction force, COP position, and trajectory during STS movement

**Fig 3:**
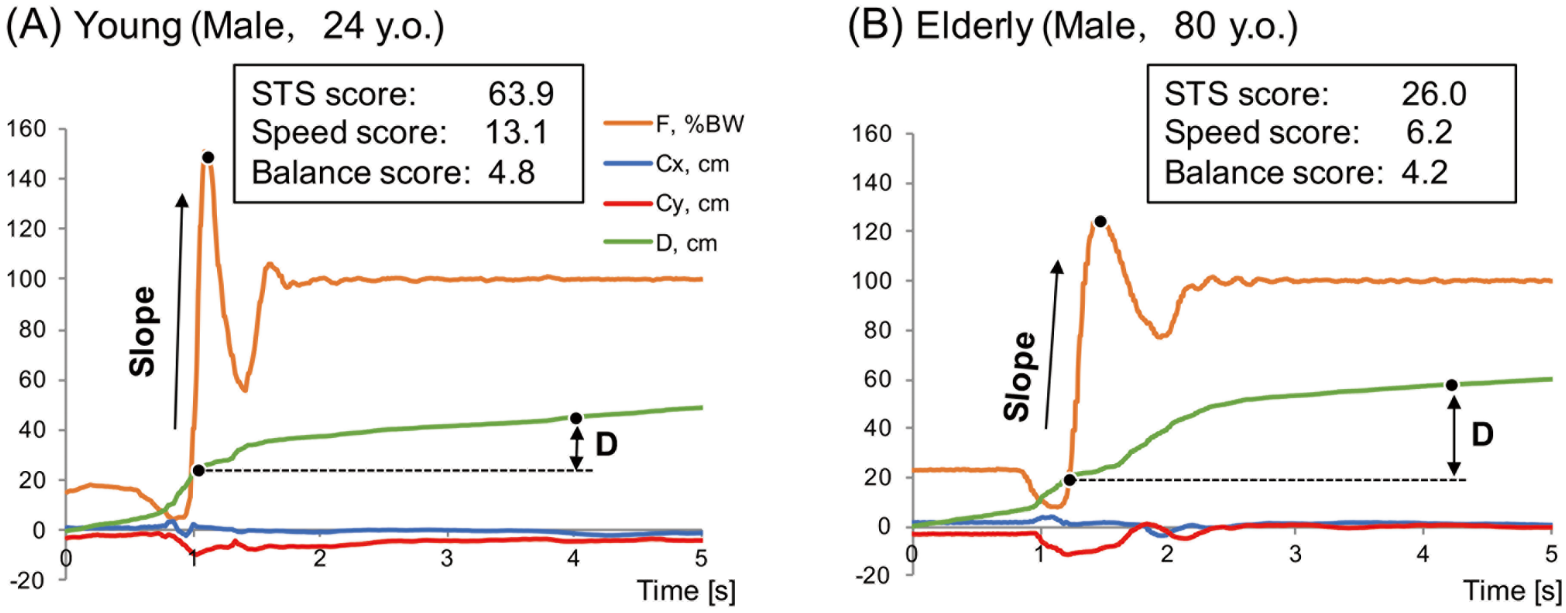
Typical graph patterns. Young (A) and elderly (B) participants in vertical reaction force, center of pressure (COP) position, and total COP distance.

During STS movement, similar curves for the vertical ground reaction force (*F*) were obtained for both typical young and elderly participants (Fig 3). Due to hip flexion, *F* decreases during the initiation of the STS movement, then reaches a peak value and oscillates around body weight. Within 3 s from the start of the STS movement, the oscillation disappeared and the total COP distance was substantially stable (not plateau). However, large differences between the typical young and elderly participants were noted in the newly defined indices.

### Age-associated changes in each index

There were significant differences in the STS indices and TUG time among seven 10-year age groups (ANOVA, *P* < 0.001). The STS score was progressively lower with increasing age (Table 2). Both STS score and TUG time changed dramatically more than at other decade changes for participants over 60 years. The mean STS score for 60s, 70s, and 80s were 77%, 68%, and 53% of that for the 20s, respectively. The mean TUG time for 70s and 80s were 123% and 143% of that for the 20s, respectively.

**Table 2:** STS, speed, and balance scores and TUG time according to 10-year age group.

Age group (years)	STS score [(ms)^-1^]	Speed score (s^-1^)	Balance score (m^-1^)	TUG time (s)
20–29	43.1 ± 12.7 ^e^^,^^f^^,^^g^	10.1 ± 3.5 ^f^^,^^g^	4.5 ± 1.0 ^c^^,^^d^^,^^e^^,^^f^^,^^g^	5.3 ± 0.5 ^d^^,^^f^^,^^g^
30–39	43.7 ± 11.0 ^d^^,^^e^^,^^f^^,^^g^	10.7 ± 2.8 ^f^^,^^g^	4.2 ± 1.1 ^d^^,^^e^^,^^f^^,^^g^	5.0 ± 0.7 ^f^^,^^g^
40–49	39.4 ± 9.7 ^e^^,^^f^^,^^g^	10.6 ± 2.6 ^f^^,^^g^	3.8 ± 0.9 ^a^^,^^g^	5.0 ± 0.6 ^f^^,^^g^
50–59	38.5 ± 9.6 ^b^^,^^f^^,^^g^	10.9 ± 2.7 ^e^^,^^f^^,^^g^	3.7 ± 1.0 ^a^^,^^b^	4.9 ± 0.7 ^a^^,^^f^^,^^g^
60–69	33.1 ± 11.0 ^a^^,^^b^^,^^c^^,^^g^	9.2 ± 2.6 ^d^^,^^g^	3.7 ± 0.9 ^a^^,^^b^	5.1 ± 0.8 ^f^^,^^g^
70–79	29.5 ± 7.9 ^a^^,^^b^^,^^c^^,^^d^^,^^g^	8.5 ± 1.9 ^a^^,^^b^^,^^c^^,^^d^^,^^g^	3.5 ± 0.7 ^a^^,^^b^	6.5 ± 1.3 ^a^^,^^b^^,^^c^^,^^d^^,^^e^^,^^g^
80–89	23.1 ± 8.6 ^a^^,^^b^^,^^c^^,^^d^^,^^e^^,^^f^	6.9 ± 2.2 ^a^^,^^b^^,^^c^^,^^d^^,^^e^^,^^f^	3.4 ± 0.7 ^a^^,^^b^^,^^c^	7.6 ± 1.4 ^a^^,^^b^^,^^c^^,^^d^^,^^e^^,^^f^
**Total**	37.0 ± 12.2	9.8 ± 3.0	3.9 ± 1.0	5.5 ± 1.2

Values are presented as mean ± standard deviation. Higher values for STS, speed, and balance scores indicate better motor performance. Shorter TUG time indicates better performance.

^a^Significant difference (p <0.05) from values of the 20s age group.

^b^Significant difference (p <0.05) from values of the 30s age group.

^c^Significant difference (p <0.05) from values of the 40s age group.

^d^Significant difference (p <0.05) from values of the 50s age group.

^e^Significant difference (p <0.05) from values of the 60s age group.

^f^Significant difference (p <0.05) from values of the 70s age group.

^g^Significant difference (p <0.05) from values of the 80s age group.

### Agreement and test–retest reliability

**Fig 4:**
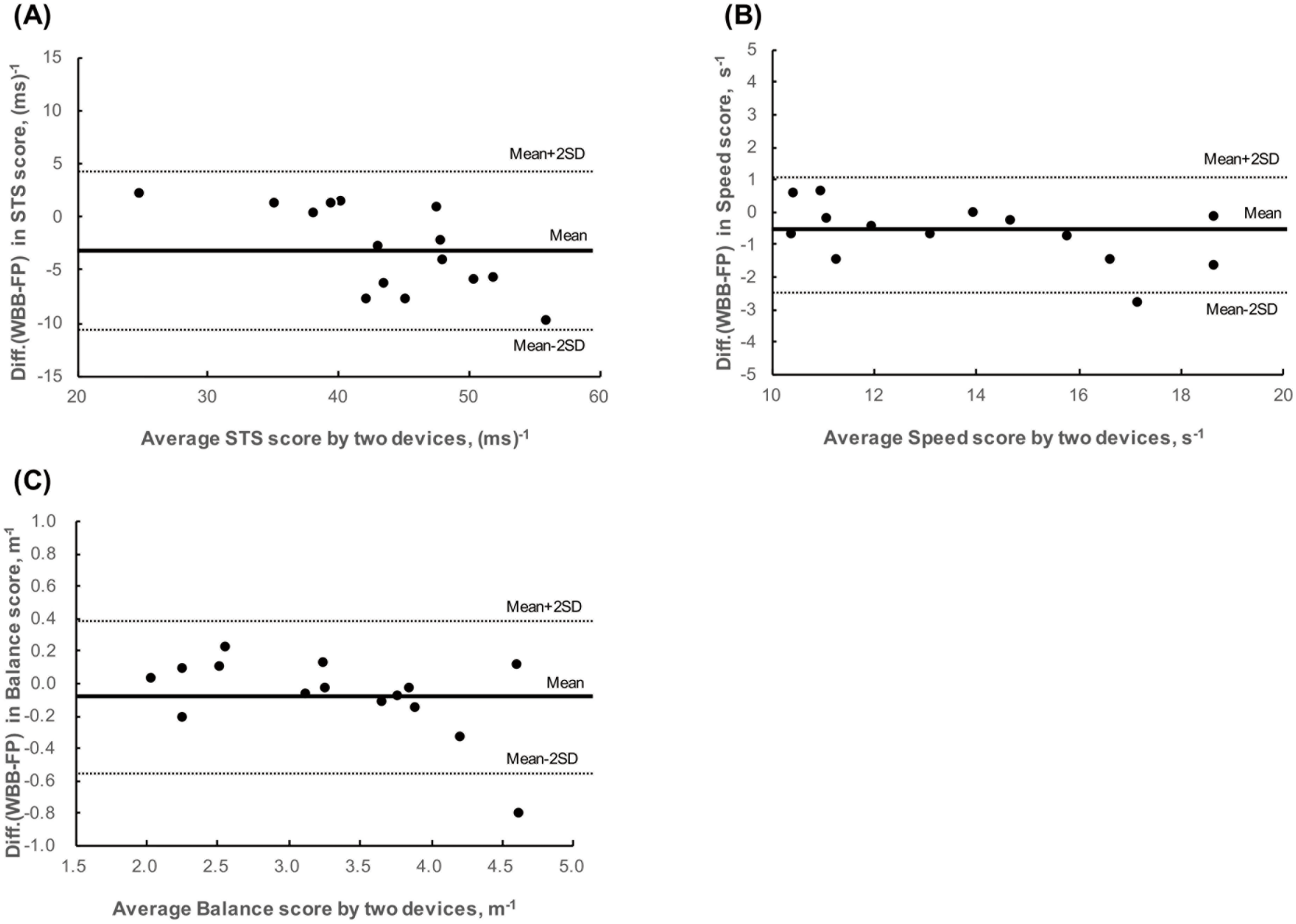
Bland–Altman plots representing comparisons between the laboratory-grade force plate (FP) and the Wii Balance Board (WBB) for STS score (A), speed score (B), and balance score (C). The mean line represents the mean difference between the devices, with the upper and lower lines representing the limits of agreement (two standard deviations).

The Bland–Altman plots for the indices are provided in Fig 4. There was no obvious relationship between the difference and the mean for each index. The plots showed negative bias for each index. The mean differences (95% limit of agreement) between WBB and FP in STS, speed, and balance scores were −3.1(−11.0 to 3.9)[(ms)^-1^], −0.7(−2.4 to 1.1) (s^-1^), and −0.1(−0.6 to 0.4) (m^-1^), respectively. Good inter-device reliabilities (ICC_(2,1)_s) were obtained for STS score [0.87, 95% confidence interval (CI) = 0.65 to 0.95], for speed score (0.96, 95% CI = 0.88 to 0.99), and for balance score (0.96, 95% CI = 0.88 to 0.99).

ICC_(1,1)_ of the STS and speed and balance scores were 0.64 (95% CI = 0.34 to 0.83), 0.78 (95% CI = 0.56 to 0.90), and 0.62 (95% CI = 0.30 to 0.81), respectively, which indicates substantial test–retest reliability.

## Discussion

The mechanically demanding STS movement changes in time as people get older, with loss of balance control and muscle strength [7, 11, 32]. In general, healthy adults perform the STS movement with small flexion of the trunk. In elderly people with muscle weakness, the STS movement is characterized by low moving speed and increased flexion of the trunk prior to rising from the seat. In the present study, we quantified the STS movement, in which the body’s center of mass is shifted upward from a sitting to a standing position without a loss of balance. Using changes in movement detected by WBB, we defined the STS score, which is based on the ground reaction force and the COP. In both young and elderly participants, the ground reaction force and the COP trajectory distance during the STS movement exhibited similar characteristic waveforms (Fig 3). However, our results demonstrate the age-related reduction in the STS score in healthy participants aged 20–88 years. Results of the TUG test revealed the age-related decrease in mobility of the participants. Furthermore, the STS score could reliably quantify the STS movement (ICC_(1,1)_ = 0.64) in the younger group. Thus, the STS score could be used to assess individual motor performance by comparing with the average value for the same 10-year age group.

We determined the STS score as a combination of the speed and balance scores with the intention to quantify performance of the STS movement. Our results show the age-related decrease in both speed and balance scores. Similarly, previous kinetic and kinematic studies have revealed differences between young and elderly people in STS movement speeds and strategies as the result of loss of balance control and muscle strength [10, 11]. Thus, both indices could represent motor performance. However, the speed and balance parameters generally conflict during the STS movement. Therefore, we used speed and balance parameters to generate a new index of estimated motor performance.

Our results show significant changes in the STS score and the TUG time over 60s. This indicates that motor performance progressively deteriorates over 60s. Previous studies have demonstrated that muscle mass and strength reach their peak value between the 20s and 40s and then decline gradually with age [2, 33–35]. Decline in knee extension torque in elderly people can compromise the capacity to perform activities, such as standing up from a chair [36]. In addition, similar to this study, our previous study has also shown a decrease in the ability of the STS movement among healthy participants >60 years of age [18].

We used WBB to measure the vertical ground reaction force and COP during the STS movement. WBB is increasingly used for assessment of postural control because it is inexpensive (<100 USD), light in weight, and portable. In addition, previous studies have examined the validity and reliability of using WBB for assessing static standing balance [24, 25]. Huurnink et al. showed that WBB yielded sufficiently accurate measurements (root-mean-square error, 0.31–0.74 mm) for the quantification of COP trajectories and overall amplitudes and velocities during single-leg stance balance tasks [25]. However, there is little information available on the accuracy of WBB as a force plate during dynamic conditions, such as STS movement. Accordingly, we compared the STS score calculated with WBB with that calculated with FP and confirmed the difference using the Bland–Altman plot (bias = −3.1 [ms]^-1^, precision = 3.9 [ms]^-1^) and inter-device reliability (ICC_(2,1)_ = 0.87). The bias error for the STS score may be attributable to the use of internally-stored calibration values for force measurements in WBB [29].

Several limitations of this study should be addressed. First, because we recruited elderly people from *rojin* clubs, the participants may have been health conscious and may have had better motor abilities than the general population. It is necessary to conduct further studies including members of the general population from defined regions. Second, we did not evaluate muscle strength (e.g., isokinetic strength) and mass in the lower extremities for comparison with the STS score. Elderly people often have weaker muscle power and torque resistance because of age-related losses in muscle mass [2, 37]. Third, the validity of the STS measurement using WBB was only examined in 5 young participants. Moreover, test–retest reliability was only examined in younger participants. Participants with a wide age range would be more appropriate for the evaluation of validity and reliability. Further study addressing these issues is now underway.

In conclusion, we developed a novel method to quantify STS movement using WBB. Notably, our results demonstrated an age-related decline in the STS score, defined as a combination of the speed and balance parameters. This quantification method of the STS movement will be useful to detect deterioration in an individual’s motor performance that would lead to difficulties in performing ADLs.
